# An experienced racial-ethnic diversity dataset in the United States using human mobility data

**DOI:** 10.1038/s41597-024-03490-y

**Published:** 2024-06-17

**Authors:** Wenfei Xu, Zhuojun Wang, Nada Attia, Youssef Attia, Yucheng Zhang, Haotian Zong

**Affiliations:** 1 Cornell University College of Architecture, Art, and Planning, Ithaca, USA; 2 Uber Technologies, San Francisco, USA; 3 Cornell University College of Arts and Sciences, Ithaca, USA; 4 University of Chicago, Chicago, USA

**Keywords:** Sociology, Geography, Social sciences

## Abstract

Despite the importance of measuring racial-ethnic segregation and diversity in the United States, current measurements are largely based on the Census and, thus, only reflect segregation and diversity as understood through residential location. This leaves out the social contexts experienced throughout the course of the day during work, leisure, errands, and other activities. The National Experienced Racial-ethnic Diversity (NERD) dataset provides estimates of diversity for the entire United States at the census tract level based on the range of place and times when people have the opportunity to come into contact with one another. Using anonymized and opted-in mobile phone location data to determine co-locations of people and their demographic backgrounds, these measurements of diversity in potential social interactions are estimated at 38.2 m × 19.1 m scale and 15-minute timeframe for a representative year and aggregated to the Census tract level for purposes of data privacy. As well, we detail some of the characteristics and limitations of the data for potential use in national, comparative studies.

## Background & Summary

Developing an empirical measure of socio-spatial dynamics and their uneven patterns is crucial for understanding how urban development policies and the built environment shapes social life. Research has shown that people’s daily activity patterns, access to places, and social interactions are shaped by current and historical urban development practices^1–3^, social networks^4–7^, residential neighborhoods^8–10^, employment opportunities^11–14^, educational context^15^, and other amenities associated with everyday activities^16,17^. Housing policies such as redlining and zoning, which entrenched racial and socioeconomic segregation^2,3,18–25^ through concentrating poor, minority residents in certain parts of the city, results in unequal school funding and school districting^13,15,26^, environmental decisions that site industrial facilities and polluting infrastructures in poor and minority neighborhoods^27,28^, amongst other impacts, all which contribute to lack of socio-spatial diversity. Despite this plethora of potential negative impacts, until recently, social science researchers have not been able to consistently measure these dynamics in both a fine-grain resolution and at scale.

**Fig. 1:**
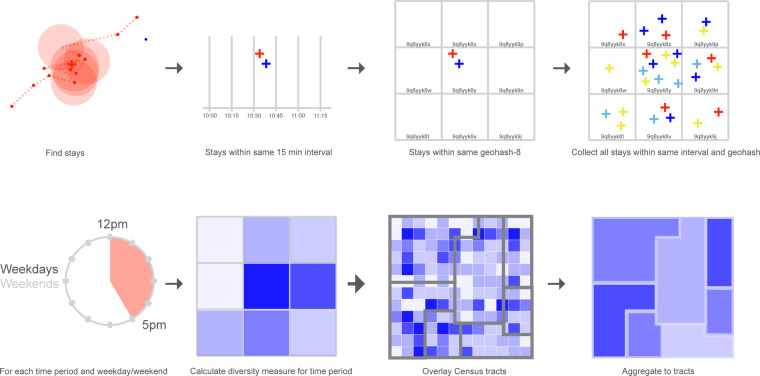
Process of deriving the NERD dataset from original smart device pings. This figure describes the steps involved in deriving the final data product. This includes the following: (1) Cluster the pings to find stays, (2) Find stays that are within 15 minutes of each other, (3) Find stays that are also within the same geohash-8 of each other, (4) Group the stays that overlap both in their 15 minute intervals and geohashes, (5) Calculate weekday/weekend and time intervals, (6) For each group of spatio-temporally overlapped stay and time interval, calculate the diversity measure at the geohash-level, (7) Overlay Census traccts, (8) Aggregate geohash-level measures to the Census tracts.

The National Experienced Racial-ethnic Diversity (NERD) dataset^29^ uses 24-hour location data for over 66 million anonymized, de-identified, and opted-in devices, compliant with the General Data Protection Regulation and the California Consumer Privacy Act, in the Spectus Clean Room platform through their *Data for Good* program (https://spectus.ai/social-impact/). Given its de-identified nature, this data has been exempted from the need to obtain Institutional Review Board approvals by our main author’s institution. We estimate 15 minute time overlaps of smart device stays in 38.2 m × 19.1 m grids across the United States in 2022. For each device, we infer probabilities of racial and ethnic backgrounds given their home Census block groups at the time of data collection, and calculate the probability of diverse social contact potential during that space and time. These measures are then aggregated to time periods of a day at the local time: “late night” (between midnight and 5:59am), “morning” (between 6:00am and 11:59am, “afternoon” (between 12:00 pm and 4:59 pm), “evening” (between 5:00 pm and 9:59 pm), and “late evening” (between 10:00 pm and 11:59 pm) as well as whether they occur on a weekday or weekend, and then to the Census tract in order to preserve privacy and develop a generalizable measure of the actual racial-ethnic diversity of a place. Figure 1 below illustrates this process.

Currently, data from the United States Census Bureau serves as the main administrative data source for reliable demographic estimates. Surveys from the Census Bureau such as the decennial census or the American Community Survey (ACS) represents residential locations updated, at best, yearly using the five-year averages from the ACS. While this data provides a crucial understanding of social mix and spatial stratification, it is not sufficiently representative of the dynamic demographic changes that might occur throughout the day as people move through a neighborhood. Moreover, its spatial proximity is only reliable at the level of the census block group and does not provide a sense of the social context *experienced* by people, which is determined by where they are in time and with whom they may cross paths. Researchers have recently begun to take advantage of increasing availability of fine-grained human mobility data to better characterize social segregation and diversity and the potential for social contact.

New human mobility data to understand social segregation and diversity and potential contact has been used in previous literature to demonstrate that experienced social context is more diverse^29,30^ and heterogeneous^31^ than previously understood through traditional means. The COVID-19 pandemic also catalyzed research on social contact^32–40^, which resulted in publicly available derivative datasets such as those describing human flows during the pandemic^33^. These studies typically look at a handful of the most populous cities or states; there has not been a study or public use dataset covering the entire United States to understand the diversity of social contexts.

We propose that the NERD dataset is a more accurate measurement of diversity than Census-based data sources as it is experienced, which we show diverges from standard measurements of diversity. We also propose a measure of diversity that only measures a hyper-locally experienced social context, unlike more traditional measurements of segregation, such as the dissimilarity index, which takes into account an areal unit’s residential racial proportions in relation to larger geographies, such as a that of a tract to a metropolitan region. This data can be used by researchers to develop a more accurate understanding of the determinants of experienced diversity; beyond research, we suggest this data can be used by policy makers to compare across geographies to understand the longer-term impacts of policies designed to encourage social mixing and access to neighborhood opportunities.

## Methods

This study uses anonymized, de-identified, and opted-in location records derived from mobile phone applications (MPA) from users that have opted-in to share data with a range of applications in categories such as games, sports, weather, and navigation, for the entire United States. The data used in this study is high-resolution location data from a collection of MPA provided through the Spectus Data Clean Room^41^. Our data comes from the weeks of March 7–20, June 6–19, September 12–25, and December 12–25, 2022. For the entire U.S. during this period, there are almost 66 million people, with stays having an average accuracy of 21 meters. MPA data has been widely applied to studies in human mobility^32,33,40,42,43^ transportation^44^, public health^45^, and urban segregation^46^. There are two intrinsic biases in the data: application usage frequency and smartphone ownership rates. The first bias is resolved by excluding unusual usage patterns. The second is more complex: According to a Pew Research Center study in 2021, while smartphone ownership is above 95% for adults in the 18–29 and 30–49 age ranges, ownership drops to 83% for adults between 50 and 64, and to 61% for adults 65 years or older. Those who have a high school education or less and those who make less than $30,000 a year also have a lower than average smartphone ownership rate of 75% and 76%, respectively. Though some argue that smartphone ownership does not significantly skew mobility estimates^47^, this study corrects population estimates by reweighting estimates using official datasets as reference. This and other sources of possible bias and validation checks are described in more detail in the “Technical Validation” section.

In addition to the main data source, we use three U.S. Census Bureau data products to evaluate the demographic profiles of devices based on their home Census tracts and block groups. The first is ACS data for 2017–2021, extracted from the National Historical GIS database^48^. Second, Metro- and Micro-politan statistical areas were extracted from the Census Bureau’s Delineation Files^49^ (https://www.census.gov/geographies/reference-files/time-series/demo/metro-micro/delineation-files.html). This dataset is used at the Census tract level to validate the residential population as sampled by the ACS in comparison to the residential population as estimated by our MPA data. The last Census Bureau dataset we use is the Longitudinal Employer-Household Dynamics (LEHD) Origin-Destination Employment Statistics (LODES) data at the block group level^50^. The LEHD program collects administrative data on employment and LODES is a data product that is annually created to provide residential and employment statistics for U.S. workers at the census block group level. It uses state-provided unemployment insurance and job earnings records to derive employment and residential locations.

### Stays and home areas

The main baseline dataset in this study are stays, areas that devices spend a significant amount of time in away from their home, derived from the underlying location pings from the MPA data using a propriety version of the Sequence Oriented Clustering algorithm^51^ (SOC). SOC is based on the Density-Based Spatial Clustering of Applications with Noise (DBSCAN) algorithm^52^ and the Ordering Points To Identify Clustering Structure (OPTICS) algorithm^53^ to identify over 43 million stays. The SOC algorithm is specific to GPS trajectories: It clusters pings of varying densities in space and time by their latitude, longitude, and timestamp, and also accounts for noise in the trajectories.

The SOC algorithm consists of the following steps: (1) For all chronologically ordered pings in a particular area, compute the geographical distance and time difference between the first and last points in a sequence *S*_*i*_. (2) Extract spatial-temporal clusters *S*_*i*_ such that $$dist\left({p}_{ia},{p}_{ia+1}\right)\le \epsilon ,\forall {p}_{i}\in {S}_{i},\,dist\left({p}_{i},{p}_{j}\right) > \epsilon $$ for any trajectory points $${p}_{i}\in {S}_{i}$$ and $${p}_{j}\in {S}_{j}$$, and $$time\left({p}_{ia},{p}_{ia+M}\right) < \tau $$ for sequence $$[{p}_{ia},{p}_{ia+1},\ldots ,{p}_{ia+M-1},{p}_{ia+M}]$$. (3) Sequences are merged together if either: two temporally consecutive sequences *S*_*i*_ and *S*_*i*+1_ have $$dist(centroi{d}_{{S}_{i}},$$$$centroi{d}_{{S}_{i+1}})\le 2\epsilon $$ and $$time\left({p}_{i,last},{p}_{i+1,first}\right) < \alpha $$ where *α* is a pre-determined minimum duration to be in transit between two stays, or two temporally consecutive sequences *S*_*i*_ and *S*_*i*+1_ have overlapping convex hulls and $$time\left({p}_{i,last},{p}_{i+1,first}\right) < \alpha $$. A convex hull is the smallest convex shape that contains all points in a collection, here the trajectory points for sequences. (4) Final sequences are pruned if end points do not satisfy condition 2). (5) False positives are removed for driving very slowly during heavy traffic and slow U-turns using a straightness and centered-distance measurement. Straightness is measured by $$\frac{dist\left({p}_{ia},{p}_{ia+M}\right)}{{\sum }_{m}dist\left({p}_{ia},{p}_{ia+m}\right)}$$ while centered-distance is measured by $$\frac{{\sum }_{m}dist\left({p}_{ia+m},centroi{d}_{{S}_{i}}\right)\left({p}_{ia+m+1}{t}_{ia+m+1}-{p}_{ia}{t}_{ia}\right)}{{\sum }_{m}\left({p}_{ia+m+1}{t}_{ia+m+1}-{p}_{ia}{t}_{ia}\right)}$$. If either measure exceed certain thresholds, they may be considered false positives.

In order to identify home areas, Cuebiq uses stays to compute a probability-based score for each possible home location based on (1) the number of days spent at a given location in the last month, (2) the daily average number of hours spent at that location, and (3) whether the time of the day spent at the location is daytime or nighttime. Each possible home area is where individuals have stayed more than two days in the past month. More days, higher average number of hours spent in an area, and spending time there during nighttime increases the score. As home areas have the possibility of changing, these calculations are created daily to account for those possible moves, whether permanent or temporary. To ensure privacy, the home areas are up-leveled to the home census block group for research purposes. Not all devices have identifiable home block groups. As such, we remove those data not associated with a home block group. A random sample of Cook County on August 1, 2022 shows around 66 percent of the total data used has a home block group. For the purposes of enhanced privacy, all calculations for the final NERD data are done at the census tract level on tracts that have more than unique 20 devices over the period of analysis. The 613 tracts for the 50 U.S. States and Washington, D.C. that have fewer than that number of unique devices are removed from the dataset.

**Fig. 2:**
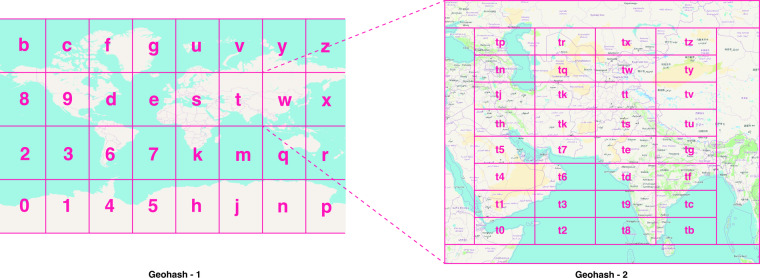
Geohash system of spatial indexes. This figure illustrates what the geohash hierarchical spatial index is. The world is first divided into a grid, with the first level containing one character in the hash, and each subsequent grid cell is further sub-divided, with each subdivided grid containing the original character of the parent hash and another character.

Stay durations as calculated as the time difference between the latest and first ping in a stay cluster and given a geohash location. Geohash is a hierarchical system of indexing space: The earth is subdivided into grids, with each grid nesting further subdivided grids. A letter or number identifier indexes the position of that grid in each “layer”. Figure 2 below shows stylized grid identifiers for geohash-1 and geohash-2.

**Fig. 3:**
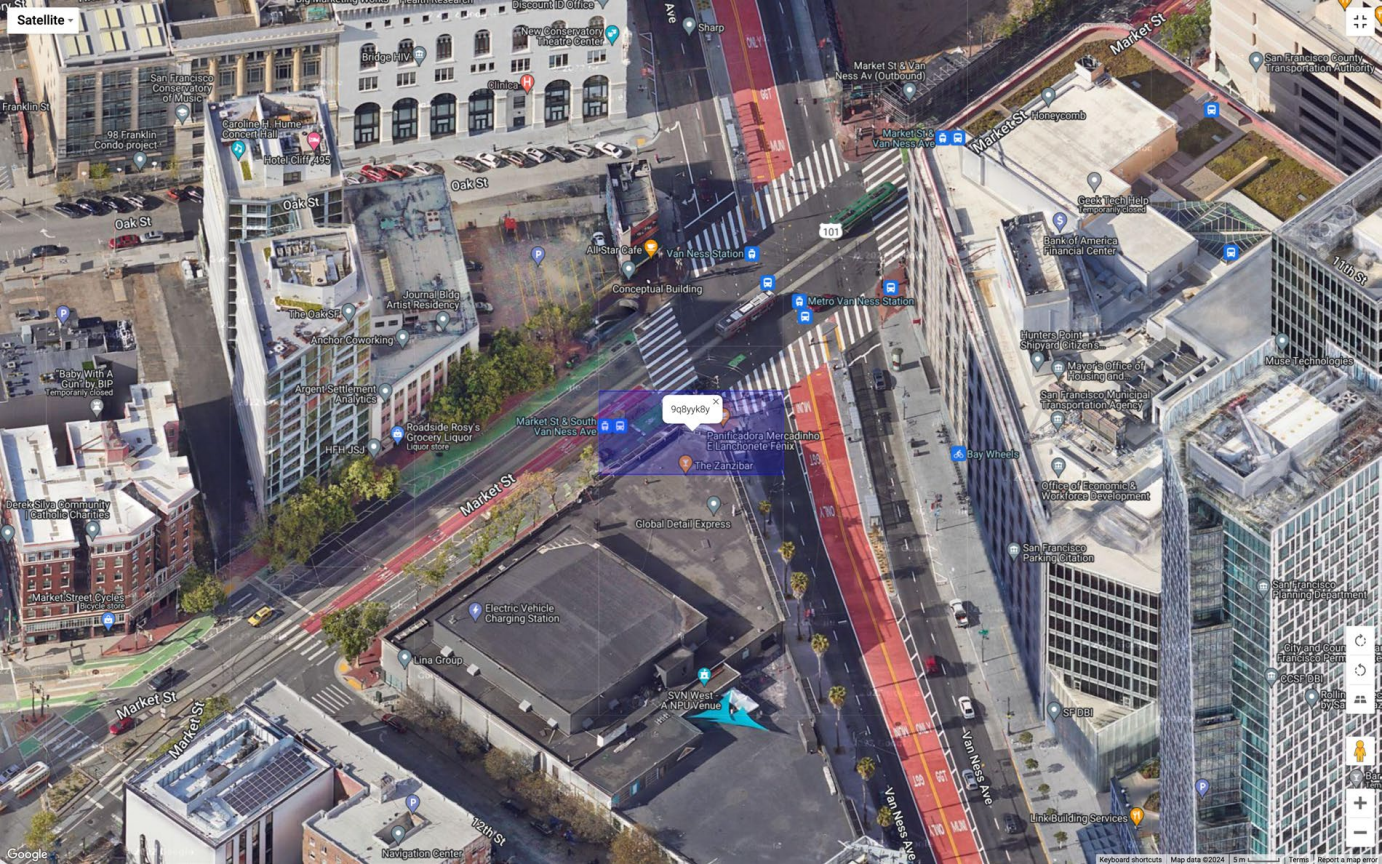
Example of geohash-8 scale from San Francisco, CA (Map data: Imagery Copyright 2024 Airbus, CNES/Airbus, Landsat/Copernicus, Maxar Technologies) d. This image shows the size of geohash-8, which is approximately the size of street-corner in San Franciso, CA.

Stays are aggregated to the geohash-8 level, which represents a 38.2 m × 19.1 m spatial resolution, our baseline unit of analysis. Figure 3 provides a sense of scale at this spatial resolution. As the geohash algorithm is a way to geocode latitude and longitude and represent location as a hierarchical unique identifier in the world, it can be faster method of conducting a spatial join or clustering multiple stays than previous methods often used with mobile phone location data such as the DBSCAN algorithm^29,54^. Moreover, it allows us to identify possible organic space-time intersections not associated with known points of interest.

**Fig. 4:**
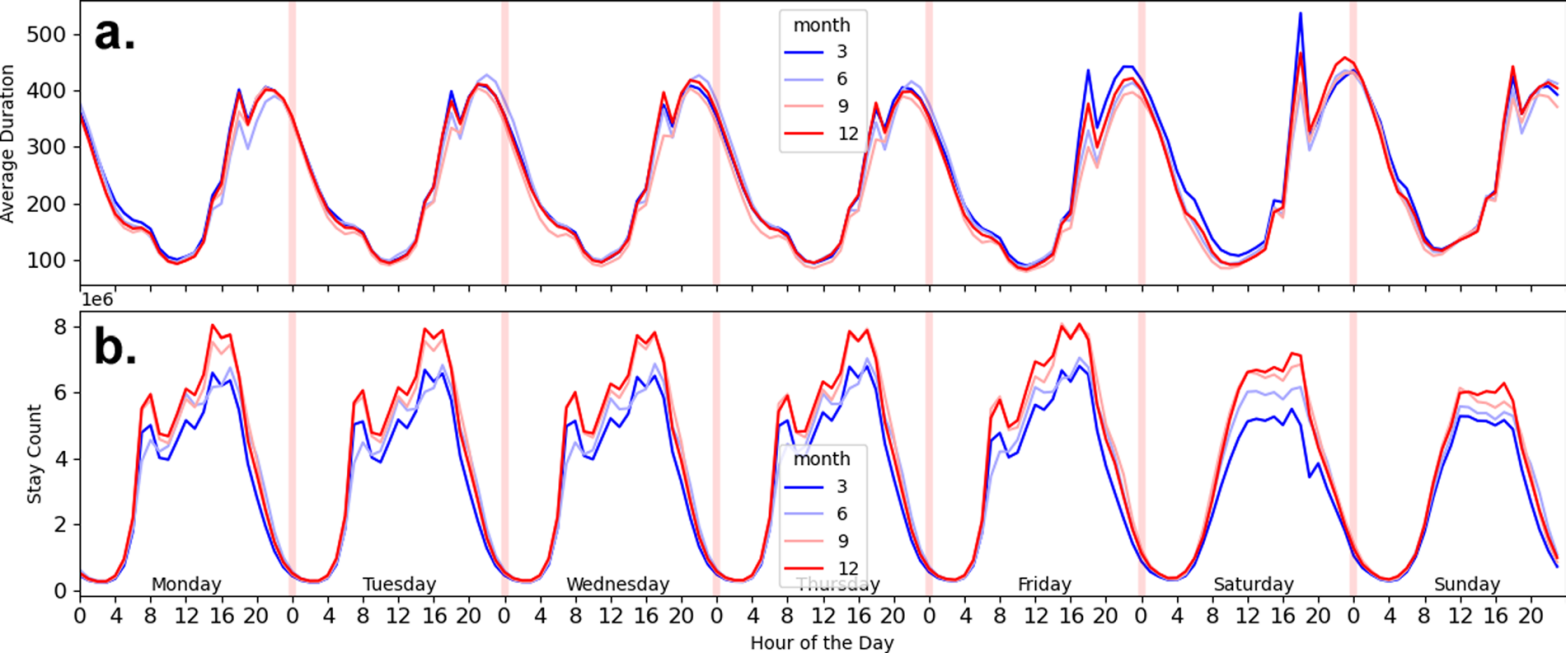
Average Stay durations (**a**) and Number of Stays (**b**) across the United States in 2022 for the months of March, June, September, and December. This figure shows two charts: The top chart (**a**), shows the average durations of the stays across days of the week and hours of the day for March (darker blue), June (light blue), September (pink), December (red). The bottom charts the average number of stays across the same time weekly period for the same months, with the same colors representing each month.

Because these data are derived from mobile devices that are typically, but not exclusively, carried on individuals, we also consider the possibility that there are devices in the data that do not represent human activity. For instance, these could be mobile devices such as tablets that may have a fixed location, but nevertheless produce pings. As such, we take two steps to remove these data: (1) We remove data where the duration of stays that are longer than 12 hours. (2) We remove data in which there are more than 20 stays at the exact latitude and longitude for a given day as another filter to detect immobile devices likely not associated with an individual. Figure 4 shows the distribution of the duration and number of stays across the country for 2022 after this filtering step. Activities, as described by the number of stays at each hour, increases during the day, with the most activity around 4 pm, while stay durations are their highest after 5 pm.

### Census tract crosswalk and population weighting

The home block groups provided by Cuebiq are in 2010 administrative boundaries. Given that the 2017–2021 American Community Survey (ACS) most closely represents the 2022 population, we use Relationship Files provided by the U.S. Census Bureau (https://www.census.gov/geographies/reference-files/time-series/geo/relationship-files.html) to crosswalk the 2017–2021 ACS data, which uses 2020 boundaries, to 2010 boundaries. Using the Relationship Files, which provides separate “parts” of all 2020 block groups, their corresponding 2010 block groups, and the area of the 2010 block group it represents. Each block group “part” is given a weight where ${w}_{p}=\frac{are{a}_{p,2020}}{are{a}_{p,2010}}$, for each “part” *p*. Then *area*_*p*,2020_ is the area that this part takes up in a 2020 Census block group and *area*_*p*,2010_ is the area that this part takes up in a 2010 Census block group. Because these are pieces of a block group, we calculate the weighted average of the 2017–2021 ACS data to 2010 boundaries based on these “parts”, such that each census block group’s values are ${b}_{2020:2010}=\frac{{\sum }_{p}^{P}{w}_{p}{b}_{2020}}{P}$ where *b*_2020_ is the 2017–2021 ACS characteristics and *P* is the number of “parts” in each 2010 boundary. We aggregate these values to the tract-level to enhance the privacy of these measures. To correct for over- or under-representation of each tract’s population, we weight each tract’s Census characteristics such that ${w}_{l}=\frac{po{p}_{Census}}{po{p}_{Cell}}$. Therefore, each tract *l*’s population is estimated by *w*_*l*_ *pop*_*Cell*_.

### Imputation of race and ethnicity membership

In order to impute demographic characteristics, we use the home tract for each device calculated for a particular day - as home locations may change throughout the year - to associate a probabilistic demographic profile for each individual based on ACS data. We use the following categories for race and ethnicity: Non-Hispanic White, non-Hispanic Black, non-Hispanic Asian, Hispanic, and non-Hispanic Other. For instance, if the tract’s population is 50% non-Hispanic White, 20% non-Hispanic Black, 10% Asian, 10% Hispanic, and 10% Other, these percentages represent the probability that the individual living in this tract belongs to each of these groups.

### Experienced diversity

At its core, our measure of experienced diversity is the probability that randomly selecting two people from an area during a certain time period will result in two individuals of different race and ethnicity memberships. We use geohash-8, which has a 38.2 m × 19 m spatial resolution as our spatial unit of analysis and use 15 minute time windows as the temporal unit of analysis. *D*_*it*_ measures the diversity in a time period and geohash.

$${D}_{it}=1-\frac{{\sum }_{j}{n}_{jit}\left({n}_{jit}-1\right)}{{N}_{it}\left({N}_{it}-1\right)}=\frac{{\sum }_{j}{n}_{jit}\left({N}_{it}-{n}_{jit}\right)}{{N}_{it}\left({N}_{it}-1\right)}$$

where *n*_*jit*_ is the number of people in group *j* ∈ *M* total groups in geohash *i* at 15-minute interval *t* and *N*_*it*_ is the total number of people in geohash-8 *i* at 15-minute interval *t*. Moreover, *D*_*it*_ can be decomposed into the experienced diversity of each racial and ethnic group ${D}_{jit}=\frac{{n}_{jit}\left({N}_{it}-{n}_{jit}\right)}{{N}_{it}\left({N}_{it}-1\right)}$. In order to calculate the diversity for longer periods we find ${\sum }_{t}{D}_{jit}$ across the number of timer periods of interest. When a stay has a long enough duration such that it overlaps with *t, t* + 1, … they will appear in multiple *D*_*jit*_. Thus, this measure thus also represents a time-weighted diversity measure, in which longer overlaps between stays will result in their inclusion into more time intervals.

### Aggregation to the census tract and time periods

In order to aggregate geohash-level measurements to the tract level (see Fig. 1), we create a weighted sum of the geohash measures by calculating how many stops are contained within each geohash-8 *i* and tract *l*. We create weights *w*_*i*_ to cross walk the geohash-level diversity measures *D*_*jit*_ to a tract-level diversity measure *D*_*jlt*_, defined by the number of stays contained in each geohash-8 and tract per day. This is in order to take into consideration that activity is heterogeneously distributed across a Census tract and, as such, certain areas of the tract might naturally experience more activity than others. Given that we are creating weights based on individual stay locations, the same geohash-8 could have different weights for different tracts when they overlap multiple tracts. However, geohash-8 is smaller than the smallest Census tract and, thus, we do not expect high proportions of membership in more than one tract.

The tract-level measures are then ${D}_{jlt}=\frac{{\sum }_{i}{w}_{i}{D}_{jit}}{{I}_{l}}$ for each period for all *I*_*l*_ geohashes in tract *l*. To calculate the final aggregate measures for each racial-ethnic group we allow ${D}_{jl}=\frac{{\sum }_{t}{D}_{jlt}}{T}$ where *T* is the total number of 15-minute intervals in each period of the day and in each day of the week, including those intervals that have no stays. *D*_*jl*_’s are averaged across weekdays and weekends for each period of the day. And finally, the tract-level diversity for each period of the day and weekday/weekend are summed across different racial-ethnic groups to get ${D}_{l}={\sum }_{j}{D}_{jl}$. Additionally, we only present measures for tracts for which there are more than 20 unique devices in the aggregate.

### Data processing

The above method is coded in Python in a Jupyter notebook on the Spectus Clean Room platform. We created an extract, transform, load (ETL) pipeline to process the data and create the NERD dataset. Because the data is indexed by the date on which it was processed our pipeline iterates over days. Within each day, we process the 1,000 most populous counties individually, given the size of the original stay data, and the other 2,142 counties in groups of 15. The populous and smaller counties are run in parallel. An incrementor indexes the counties and dates to be processed. Each run of a populous county or a group of 15 counties took approximately 2–20 minutes to complete the entire data processing on the 4 nodes with 8 CPUs and 64 GB RAM per node.

## Data Records

The NERD data product is a U.S. Census tract level dataset of experienced diversity for representative weeks from 2022 using 2010 tract boundaries and 2021 5-Year ACS. Along with these measures, we include accompanying Census demographic and socioeconomic data and residential diversity measures from the 2021 5-year ACS that have been cross-walked to 2010 Census boundaries in order to facilitate the census comparison process. Experienced diversity is presented in its total (“total_diversity_exp”) and composite metrics (“white_diversity_exp”, “black_diversity_exp”, “asian_diversity_exp”, “hispanic_diversity_exp”, “other_diversity_exp”). The accompanying Census data are made available for two reasons: The first is to allow users to easily compare the experienced diversity data to standard calculations such as residential diversity and Census characteristics. The second is that the Cuebiq mobility data is only available at the 2010 Census block group level; therefore, we provide the cross-walked Census data for 2021 5-year ACS with 2010 boundaries. The Census data is from the IPUMS National Historical Geographic Information System database^48^. A copy of the data can be downloaded from the Open Science Framework^55^. The data is separated by state for ease of download in the format *diversity_intervals_[STATE ABBREVIATION].geojson*. Each file is in the GeoJSON geospatial data format. A CSV for each file also been added in the format *diversity_intervals_[STATE ABBREVIATION].csv*. These files exclude the geometries for reduce the size of the file. Below is the data dictionary:

- STATEFP10: 2010 Census state FIPS code
- COUNTYFP10: 2010 Census county FIPS code
- TRACTCE10: 2010 Census tract FIPS code
- GEOID10: Census tract identifier; a concatenation of 2010 Census state FIPS code, county FIPS code, and census tract code
- NAME: Full name of the Core Based Statistical Area
- total_pop: Total Population in 2020
- white_perc: Percentage Non-Hispanic White Alone in 2020
- black_perc: Percentage Non-Hispanic Black Alone in 2020
- indigenous_perc: Percentage Non-Hispanic American Indian and Alaska Native alone in 2020
- asian_perc: Percentage Non-Hispanic Asian alone in 2020
- pac_isl_perc: Percentage Non-Hispanic Native Hawaiian and Other Pacific Islander Alone in 2020
- other_perc: Percentage Non-Hispanic Some Other Race Alone in 2020
- two_more_perc: Percentage Non-Hispanic Two or More Races in 2020
- hispanic_perc: Percentage Hispanic in 2020
- total_scho: Population 25 years and over in 2020
- ba_perc: Percentage Bachelor’s Degree in 2020
- ma_perc: Percentage Master’s Degree in 2020
- prof_perc: Percentage Professional School Degree in 2020
- phd_perc: Percentage Doctorate Degree in 2020
- ba_higher_perc: Percentage Bachelor’s Degree or Higher in 2020
- median_inc: Median household income in the past 12 months (in 2021 inflation-adjusted dollars)
- white_diversity_exp: White Experienced Diversity in 2022
- black_diversity_exp: Black Experienced Diversity in 2022
- asian_diversity_exp: Asian Experienced Diversity in 2022
- hispanic_diversity_exp: Hispanic Experienced Exposure in 2022
- other_diversity_exp: Some Other Race Alone Experienced Diversity in 2022
- total_diversity_exp: Total Experienced Diversity in 2022
- total_diversity_resi: Residential Diversity Based on 2020 Census
- diff: Difference in Percentage between Total Experienced Diversity and Residential Diversity
- interval: The time period of the day: “late night” (between midnight and 5:59am), “morning” (between 6:00am and 11:59am, “afternoon” (between 12:00 pm and 4:59 pm), “evening” (between 5:00 pm and 9:59 pm), and “late evening” (between 10:00 pm and 11:59 pm)
- weekday: “weekday”’ for Mondays through Fridays, “weekend” for Saturdays through Sundays

## Technical Validation

Because the underlying cell phone data is unintentionally collected, we assume that it has different sources of bias and uncertainty, including bias in representation of the United States population and uncertainty in the temporal and geographic variation of this representation. In order to validate the NERD dataset for public use, we look at several bias, sensitivity, and heterogeneity checks of the underlying data. Additionally, we compare it to the Census population to check night-time populations and the patterns of work-related stays to the Longitudinal Employer-Household Dynamics in 2019 and 2020 to test the populations during the day.

### Comparison to census demographics

We find the Pearson correlation between the original, before de-biasing, MPA sample population density in 2022 and the Census population density is 81.8% while the Spearman correlation coefficient is 93.4% for the whole country, which is similar or higher to previous studies^56–58^. Analyses for years prior to 2022 show similar results.

### Sensitivity analysis of geohash and time bucket

**Fig. 5:**
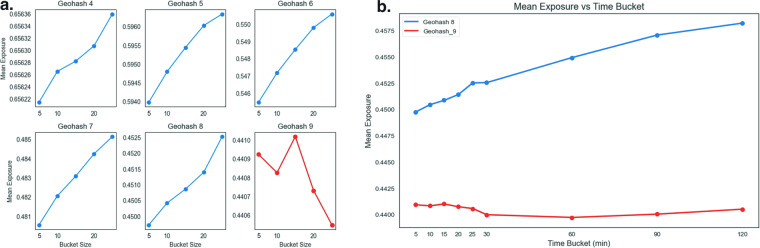
Geohash spatial resolution tests. This figure shows: For chart (**a**), diversity levels going up as diversity is calculated on using larger time buckets, from 5 min to 30 minute for geohash 5–8, while we see diversity going down in larger time bins for geohash 9; for chart (**b**), a comparison with diversity increasing for time buckets from 5–120 minutes in geohash 9, while geohash 9 is largely decreasing or flat during the same time period.

We perform resolution and time bucket sensitivity analyzes to optimize our spatial unit of analysis. Figure 5a uses a sample of stays from Cook County, IL in August 1, 2022 and shows that larger geohashes result in higher mean diversity scores, as these are larger units that would capture more stays within each unit. For instance, at geohash 4, the diversity in Cook County ranges from 0.6562–0.6563 for time periods between 5–30 minutes while for geohash 8 the diversity is around 0.44955–0.4525. Diversity also increases with larger time buckets, which would also capture more stays within that time unit, with a range between 0.45–0.4575 between 5 and 120 minute buckets.

However, the behavior of geohash-9 and beyond do not follow the patterns of larger resolution geohash units as geohash-9 is 4.8 m × 4.8 m. Figure 5b below shows that at geohash-9, longer time buckets do not exhibit a clear upward pattern in diversity. One possibility here is that since geohash-9 is smaller than the median accuracy of the stay data, a stay’s true location occurs in a random selection of geohash units around the actual location of the stay. Our choice of geohash-8 is premised on these studies that suggest it is the smallest possible unit of analysis.

### Geographic variation

**Fig. 6:**
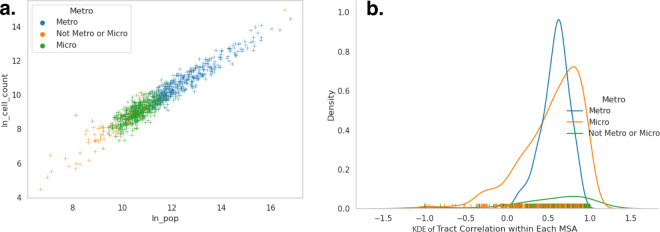
Scatterplot of CBSA (**a**) and distribution of tract-level correlation (**b**) for Log Population vs. Log MPA Counts per CBSA (Note: As Kernel Density Estimation (KDE) approximates density, it extends beyond 1, while rug shows density of correlation ranges from [−1, 1]). This figure shows: In chart (**a**), a scatterplot of CBSA-level natural-log population count on the x-axis and the natural-log of the original cell phone stay counts on the y-axis. The charts shows a broadly linear relationship. The colors of each point in the scatterplot are blue for CBSAs labeled Metropolitan, green for CBSAs labeled Micropolitan, and orange for CBSAs that are too small for either label. In chart (**b**), there are three kernel-density estimates (KDE) of the distribution of within-CBSA travel-level correlations between the natural-log population count and the natural-log of the original cell phone stay counts, with each KDE colored using the same color scheme as chart (**a**).

Another concern with a dataset with a national scope is the possible heterogeneity of representativeness, given that smart device usage could have an urban bias. We associate each tract with its Metropolitan or Micropolitan membership, or lack thereof. Looking at the across-Core Based Statistical Area (CBSA) correlation between the Census and the original MPA data population, before we correct for biases, we can see that the relationship is linear on a log-log scale, with a Pearson correlation of 0.957, regardless of the CBSA type, as shown by Fig. 6a. Moreover, when we do a within-CBSA comparison of the population to MPA data population for different types of CBSAs at the tract level(see Fig. 6), the most frequent correlation in each group is higher for Micropolitan and smaller areas (see Fig. 6b): it is 0.65 for ‘Not Metro or Micro’ areas, 0.63 for Micropolitan areas, and 0.6 for Metropolitan areas. There is more variability in the CBSA correlations with smaller populations, but overall, these results show that, compared to the Census population, the MPA data is also surprisingly consistent geographically in terms of its night-time population representation.

### Comparison to employer-household dynamics

Work-home pairs reflect the dynamics of commuting behaviors. Similar to home block group areas, the MPA data also identifies the work block group areas based on cell phone users’ trajectories, using a similar method of home area identification, but looking at the most common and frequent locations during the day. Cell phone users with both home and work block groups identified by the SOC algorithm are compared here with the work-home pairs from the Longitudinal Employer-Household Dynamics Origin-Destination Employment Statistics (LODES).

**Fig. 7:**
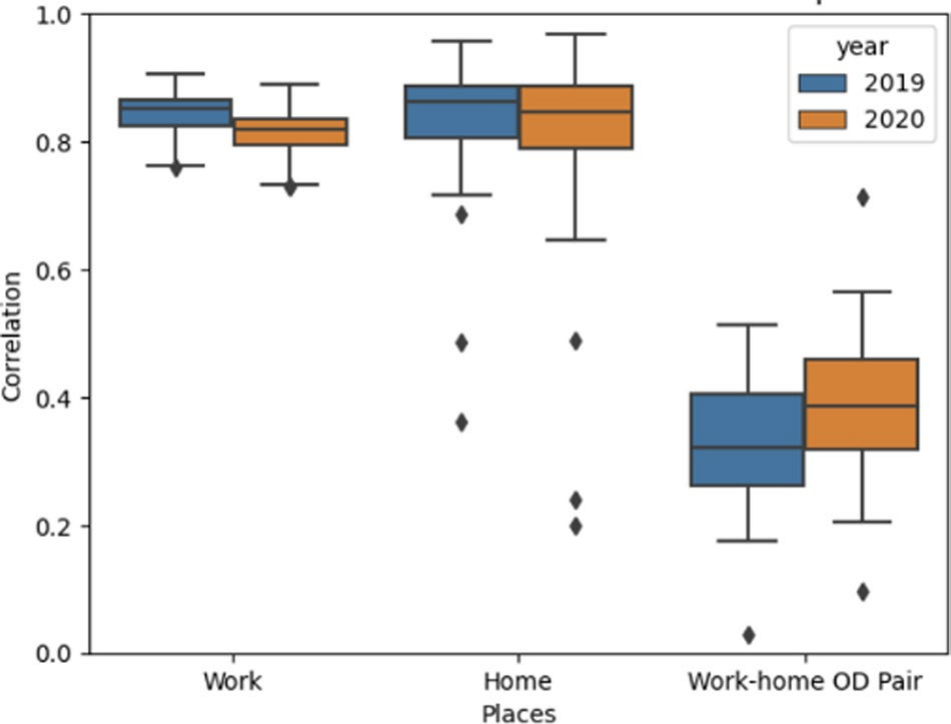
Pearson correlation between LODES and cell phone data at the block group level by state. This figure shows the Pearson correlation at the block group-level between the cell phone data population counts and the LEHD Origin-Destination Employment Statistics for work, home, and the work-home origin-destination pair by state. Comparisons in 2019 are shown in blue and those for 2020 are down in orange.

Given the impact of COVID-19 on human commuting behaviors, we focus on comparing the MPA data and LODES work-home pairs in 2019 (before pandemic) and 2020 (during pandemic), as another layer of temporal validation. We retrieve the daily average workers on weekdays of the second full week in March, June, September, and December for 2019 and 2020. Figure 7 below compares the home, work, and origin-destination pairs between the cell phone and LODES datasets in 2019 and 2020 by state. Correlations are over 0.8 when we compare either home locations or work locations regardless of year, while the correlations drop to 0.35 if we compare workers with both work and home locations matched, as both the LODES and MPA origin and destination matrices are fairly sparse. Here, the OD pair refers to what both LODES and the MPA data have estimated to be the “work” and “home” block groups of a device. During the pandemic, the correlations at either home or work locations are slightly lower than before. The correlation decrease is likely due to the fact that many workers can either move to other dwellings or work from home, but LODES keeps using the registered homes and offices from state and federal records.

## Usage Notes

In this section, we provide a description of the data product and address some of the limitations of the current version of this data.

**Fig. 8:**
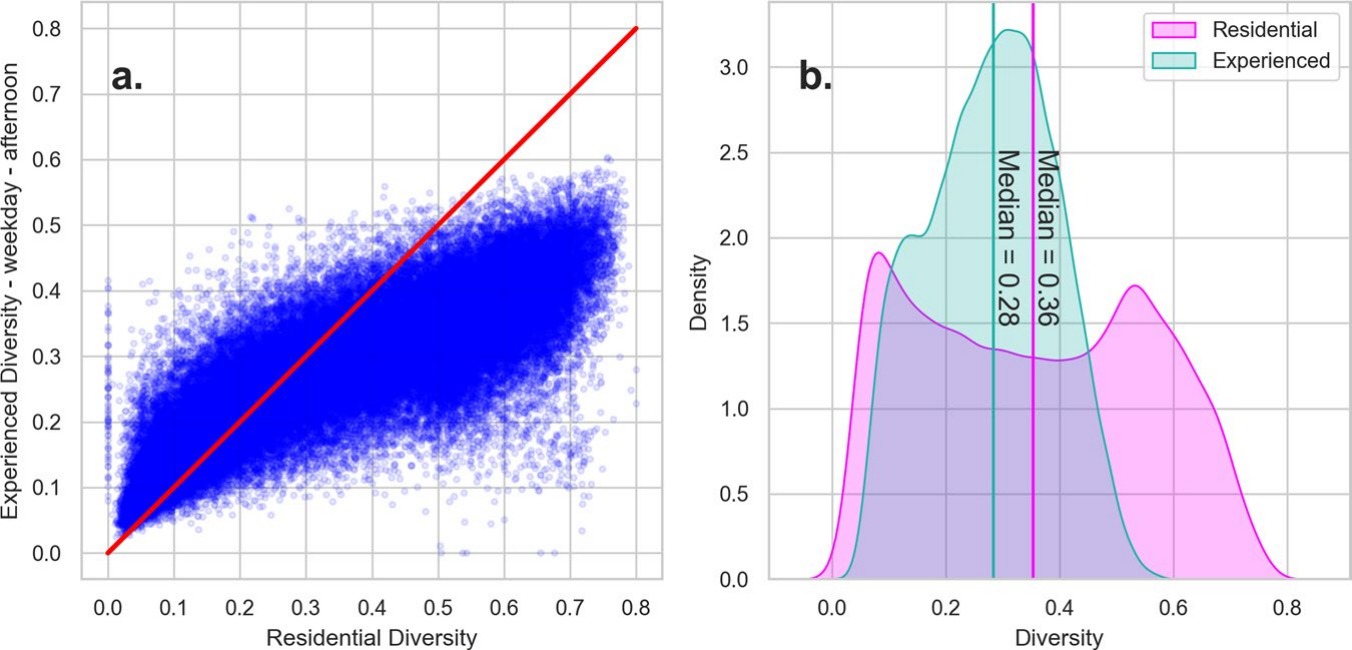
Residential and Experienced diversity scatterplot (**a**) and distribution (**b**) for weekday afternoons. This figure shows: In chart (**a**), a scatterplot between the residential and experienced diversity measures for weekday afternoons, with a red line showing the one-to-one ratio, and in chart (**b**), the distribution of the experienced diversity in turquoise and the residential diversity in pink, with median lines colored in the same way.

Figure 8 shows a comparison between the NERD and residential diversity measures for a representative weekday afternoon. In Fig. 8a, we plot each tract-level residential diversity for the entire country against the NERD measure. The Supplementary Information shows these plots for all time periods.

**Fig. 9:**
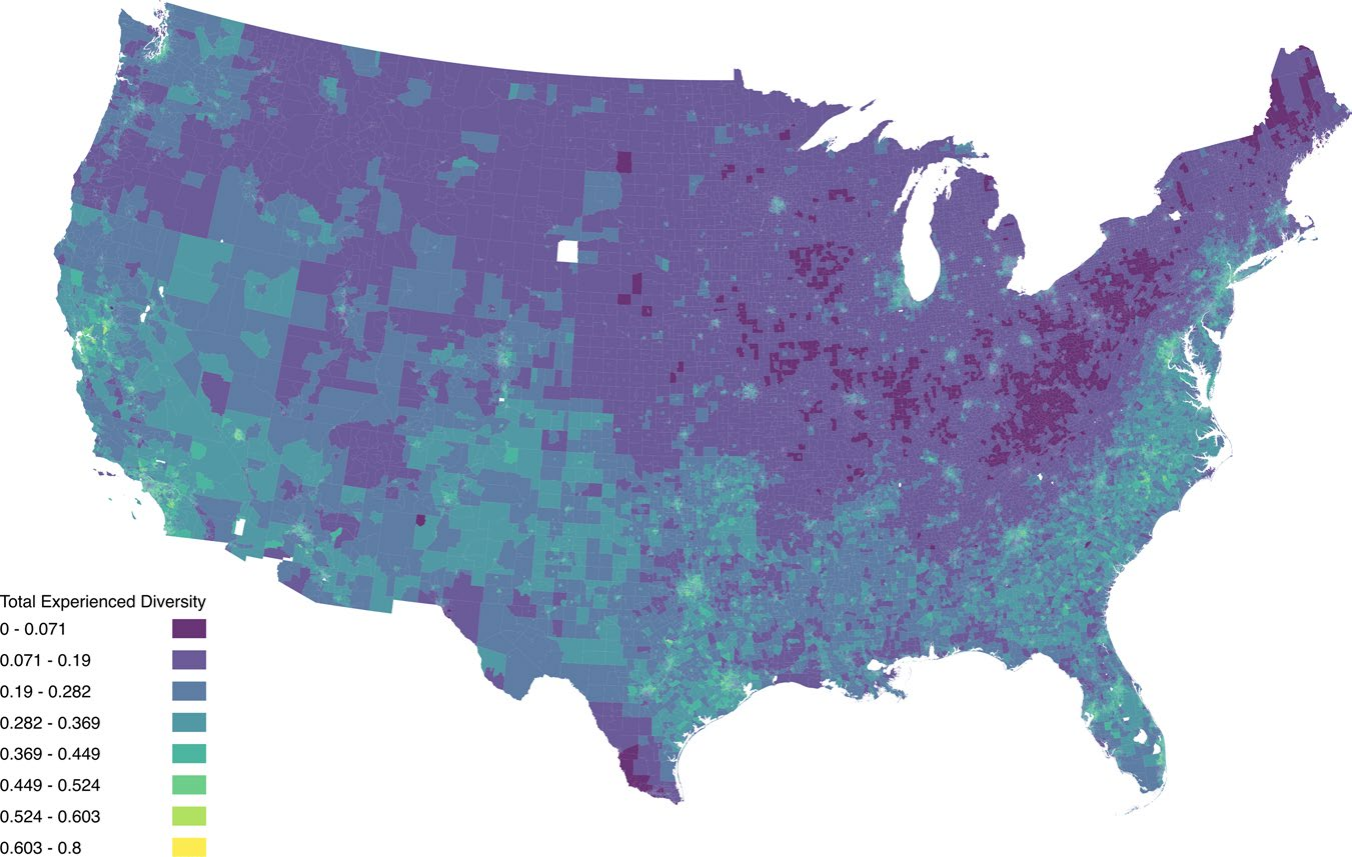
Total Experienced Racial and Ethnic Diversity for Weekday Afternoons. This map shows a choropleth of the experienced diversity for weekday afternoons across the US at the Census tract level. The bins are 0–0.071, 0.071–0.19, 0.19–0.282, 0.282–0.369, 0.369–0.449, 0.449–0.524, 0.524–0.603, 0.603–0.8.

Figure 9 shows provides a choropleth map of experienced diversity. Note that tracts missing diversity measures in this dataset are due to lack of large enough sample size of 20 unique individuals within the space and time bounds) in the data.

Table 1 shows the mean, standard deviation, minimum, maximum, and percentile breakdowns of each measure by their time and weekday/weekend intervals. It should be noted that all individual race and ethnicity diversity measures have a theoretical range of 0–0.25 while total diversity measures have a theoretical range of 0–0.8.

**Table 1:** Experienced Diversity by Race and Ethnicity Breakdown.

		Weekend	Weekend	Weekend	Weekend	Weekend	Weekend	Weekend	Weekend	Weekend	Weekend
		12 am – 5:59 am	6 am – 11:59 am	12 pm – 4:59 pm	5 pm – 9:59 pm	10 pm - 11:59 pm	12 am – 5:59 am	6 am – 11:59 am	12 pm – 4:59 pm	5 pm - 9:59 pm	10 pm -11:59 pm
Count		72444	72444	72444	72444	72444	72402	72402	72402	72402	72799
Total Div. Exp	mean	0.049	0.236	0.278	0.214	0.082	0.068	0.208	0.288	0.240	0.092
	std	0.041	0.098	0.111	0.098	0.056	0.053	0.089	0.112	0.103	0.064
	min	0.000	0.000	0.000	0.000	0.000	0.000	0.000	0.000	0.000	0.000
	25%	0.022	0.160	0.193	0.137	0.040	0.029	0.139	0.202	0.160	0.043
	50%	0.040	0.239	0.283	0.209	0.069	0.055	0.207	0.293	0.240	0.079
	75%	0.065	0.310	0.361	0.285	0.110	0.092	0.273	0.372	0.318	0.126
	max	0.589	0.563	0.603	0.577	0.492	0.579	0.650	0.621	0.585	0.519
White Div. Exp	mean	0.018	0.091	0.107	0.081	0.030	0.024	0.080	0.111	0.091	0.033
	std	0.012	0.034	0.039	0.033	0.018	0.016	0.032	0.039	0.035	0.021
	min	0.000	0.000	0.000	0.000	0.000	0.000	0.000	0.000	0.000	0.000
	25%	0.009	0.064	0.077	0.055	0.016	0.012	0.055	0.081	0.064	0.018
	50%	0.015	0.093	0.111	0.081	0.026	0.021	0.081	0.115	0.093	0.030
	75%	0.023	0.117	0.137	0.106	0.040	0.033	0.104	0.141	0.118	0.045
	max	0.172	0.191	0.214	0.201	0.144	0.176	0.250	0.213	0.209	0.149
Black Div. Exp.	mean	0.009	0.041	0.048	0.038	0.015	0.013	0.036	0.050	0.043	0.017
	std	0.009	0.032	0.037	0.031	0.015	0.014	0.028	0.038	0.034	0.018
	min	0.000	0.000	0.000	0.000	0.000	0.000	0.000	0.000	0.000	0.000
	25%	0.002	0.014	0.017	0.013	0.004	0.003	0.013	0.019	0.015	0.004
	50%	0.005	0.032	0.038	0.028	0.009	0.007	0.027	0.039	0.032	0.010
	75%	0.013	0.061	0.073	0.056	0.020	0.017	0.053	0.075	0.064	0.023
	max	0.096	0.165	0.187	0.165	0.137	0.172	0.159	0.205	0.179	0.152
Asian Div. Exp	mean	0.005	0.024	0.028	0.022	0.009	0.007	0.022	0.030	0.025	0.010
	std	0.010	0.026	0.030	0.026	0.014	0.013	0.024	0.031	0.028	0.016
	min	0.000	0.000	0.000	0.000	0.000	0.000	0.000	0.000	0.000	0.000
	25%	0.001	0.007	0.008	0.006	0.002	0.001	0.006	0.009	0.007	0.002
	50%	0.002	0.014	0.017	0.013	0.004	0.003	0.013	0.018	0.015	0.004
	75%	0.005	0.030	0.035	0.027	0.010	0.008	0.027	0.037	0.031	0.011
	max	0.198	0.173	0.193	0.195	0.173	0.196	0.170	0.195	0.189	0.177
Hispanic Div Exp.	mean	0.012	0.058	0.068	0.053	0.021	0.017	0.051	0.070	0.059	0.023
	std	0.012	0.039	0.045	0.038	0.019	0.017	0.035	0.046	0.041	0.021
	min	0.000	0.000	0.000	0.000	0.000	0.000	0.000	0.000	0.000	0.000
	25%	0.004	0.025	0.030	0.021	0.006	0.005	0.022	0.031	0.025	0.007
	50%	0.008	0.048	0.057	0.042	0.014	0.011	0.042	0.059	0.048	0.016
	75%	0.017	0.089	0.104	0.079	0.029	0.024	0.076	0.106	0.089	0.034
	max	0.113	0.190	0.206	0.194	0.143	0.144	0.193	0.211	0.191	0.151
Other Div Exp.	mean	0.005	0.022	0.026	0.020	0.008	0.006	0.020	0.027	0.022	0.009
	std	0.008	0.012	0.014	0.013	0.008	0.009	0.011	0.014	0.013	0.009
	min	0.000	0.000	0.000	0.000	0.000	0.000	0.000	0.000	0.000	0.000
	25%	0.002	0.015	0.018	0.013	0.004	0.003	0.013	0.019	0.015	0.004
	50%	0.003	0.020	0.023	0.018	0.006	0.005	0.017	0.024	0.020	0.006
	75%	0.006	0.026	0.031	0.024	0.009	0.008	0.023	0.032	0.026	0.011
	max	0.177	0.160	0.176	0.190	0.150	0.178	0.165	0.200	0.170	0.155
Total Div Resi.	mean	0.355	0.355	0.355	0.355	0.355	0.355	0.355	0.355	0.355	0.356
	std	0.202	0.202	0.202	0.202	0.202	0.202	0.202	0.202	0.202	0.202
	min	0.000	0.000	0.000	0.000	0.000	0.000	0.000	0.000	0.000	0.000
	25%	0.172	0.172	0.172	0.172	0.172	0.172	0.172	0.172	0.172	0.172
	50%	0.352	0.352	0.352	0.352	0.352	0.352	0.352	0.352	0.352	0.353
	75%	0.531	0.531	0.531	0.531	0.531	0.531	0.531	0.531	0.531	0.532
	max	0.800	0.800	0.800	0.800	0.800	0.800	0.800	0.800	0.800	0.800

**Fig. 10:**
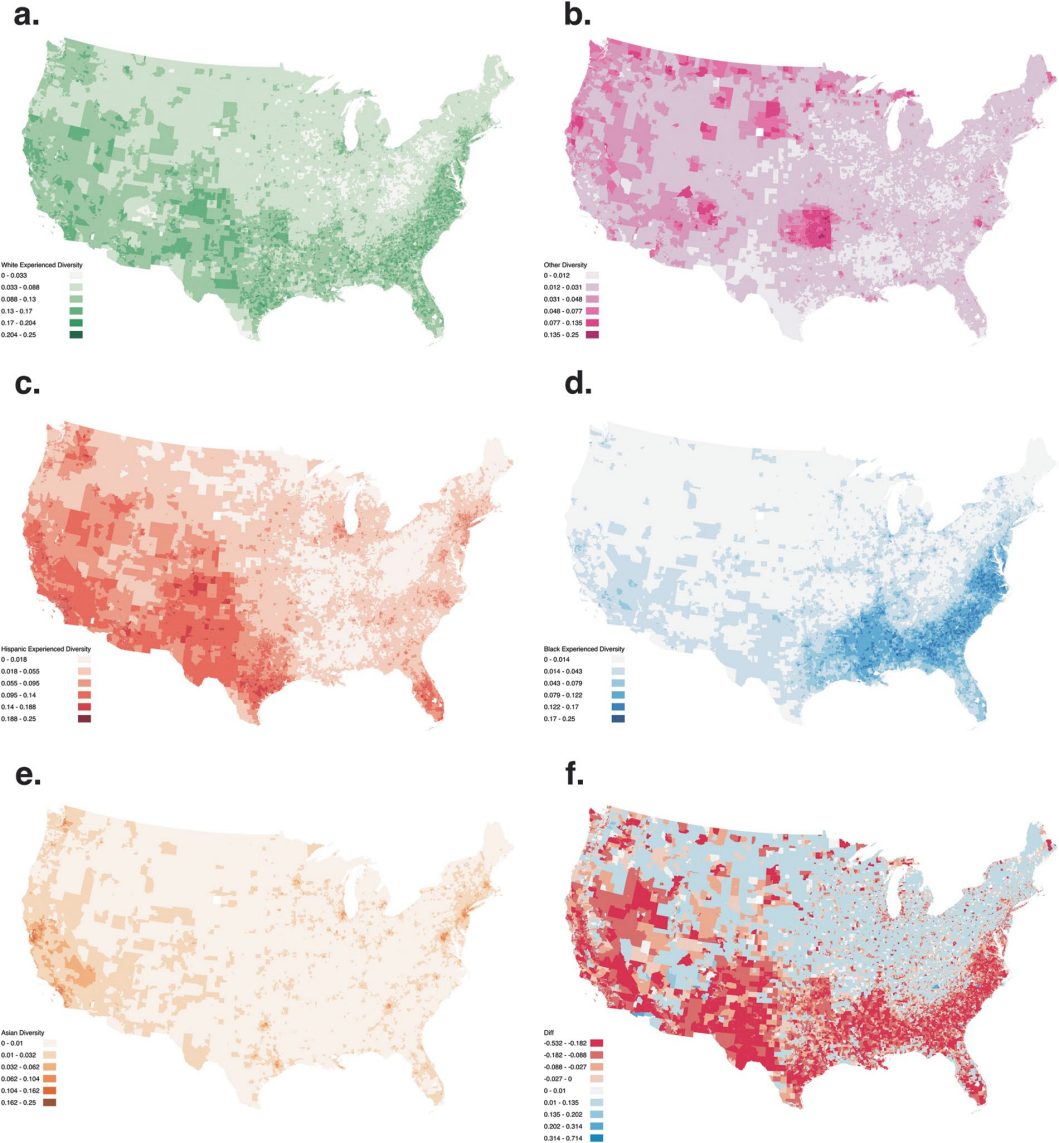
Experienced Diversity by Race for Weekday Afternoons: White (**a**), Other (**b**), Hispanic (**c**), Black (**d**), Asian (**e**), and Difference between the Experienced and Residential Diversity (f). The choropleth ranges from dark blue to yellow. This figure shows choropleths of the experienced diversity for weekday afternoons for White (**a**) population with bins 0–0.033, 0.033–0.088, 0.088–0.13, 0.13–0.17, 0.17–0.204, 0.204–0.25 ranging from light green to dark green, Other (**b**) population with bins 0–0.012, 0.012–0.031, 0.031–0.048, 0.048–0.077, 0.077–0.135, 0.135–0.25 ranging from light pink to dark magenta, Hispanic (**c**) population with bins 0–0.018, 0.018–0.055, 0.055–0.095, 0.0955–0.14, 0.14–0.188, 0.188–0.25 ranging from light orange to dark orange, Black (**d**) population with bins 0–0.014, 0.014–0.043, 0.043–0.079, 0.079–0.122, 0.122–0.17, 0.17–0.25 ranging from light blue to dark blue, Asian (**e**) population with bins 0–0.01, 0.01–0.032, 0.032–0.062, 0.062–0.104, 0.104–0.162, 0.162–0.25 ranging from light brown to dark brown, and difference between the Experienced and Residential Diversity (**f**) with bins −0.532–0.182, −0.182–0.088, −0.088–0.027, −0.027−0, 0–0.01, 0.01–0.135, 0.135–0.202, 0.202–0.314, 0.314–0.714 ranging from red to blue.

Figure 10 shows the experienced diversity measure decomposed by race and ethnicity breakdown for White, Asian, Hispanic, Black, and Other Single Race, as well as the difference between the total experienced versus residential diversity.

**Fig. 11:**
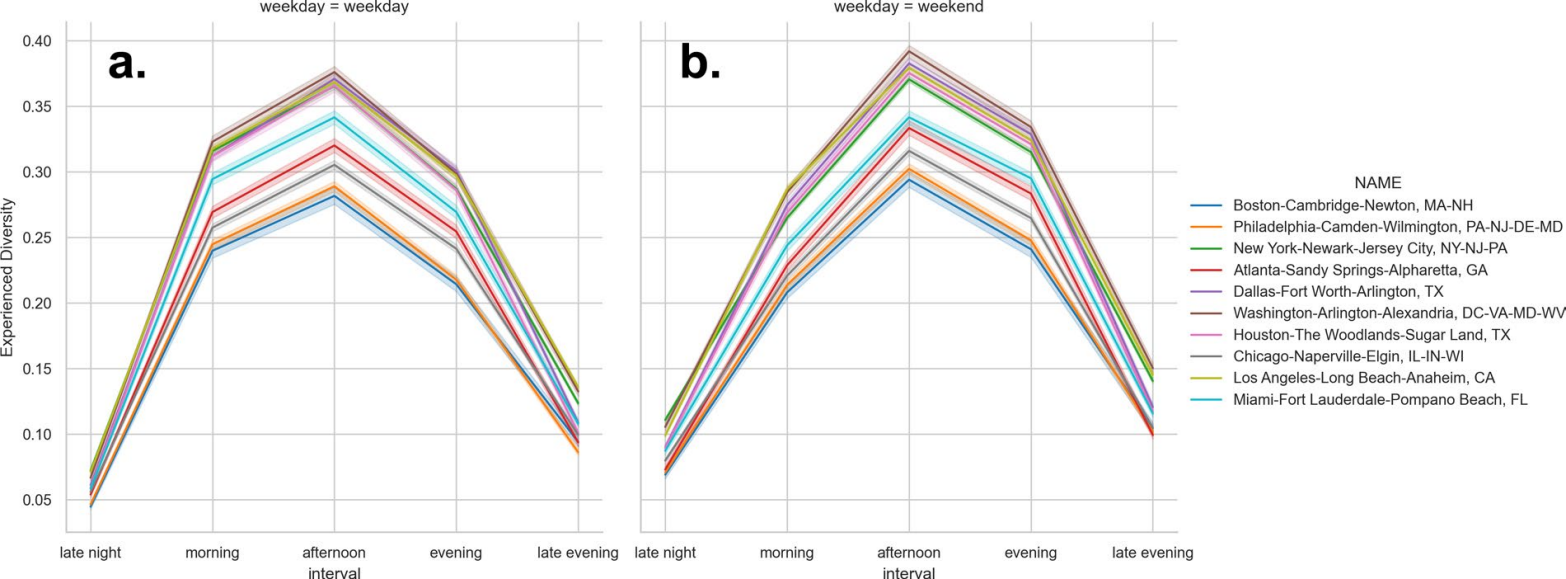
Total Experienced Diversity by Top 10 CBSAs. Levels of experienced diversity for weekday (**a**) and weekend (**b**) for late morning, morning, afternoon, evening, and late evening for the top 10 CBSAs: Boston-Cambridge-Newton, MA-NH, Philadelphia-Camden-Wilmington, PA-NJ-DE-MD, New York-Newark-Jersey City, NY-NJ-PA, Atlanta-Sandy Springs -Alpharetta, GA, Dallas-Fort Worth-Arlington, TX, Washington-Arlington-Alexandra-, DC-VA-MD-WV, Houston-The Woodlands-Sugar Land, TX, Chicago-Naperville-Elgin, IL-IN-WI, Los-Angeles-Long Beach-Anaheim, CA, Miami-Fort Lauderdale-Pompano Beach, FL.

Figure 11 shows the variation in experienced diversity by period of the day for weekdays and weekends for the ten most populous CBSAs, which corresponds to Fig. 4 showing the most activity in the afternoons.

There are certain limitations to the current version of this data product due to the need to preserve privacy and to establish ease of use through the aggregation of measures, the continued research efforts in this area to validate this novel form of big data, the neighborhood- versus people-centric nature of the NERD data product, and issues of margins of error possibly influencing the measurement of diversity measures. Future iterations of this data product will aim to address these questions.

First, the original measurements that we calculated are at a 15 minute time interval and geohash-8 level, though we aggregated the data across time and to the level of the Census tract. This is to allow these measures to be easily compared to standard measures of diversity and other demographic characteristics, which are generally using Census administrative boundaries. The second reason for lowering the public-facing spatial and temporal resolution of our data to the tract level is to preserve privacy: Not only do we filter out those tracts that do not have more than 20 unique devices to ensure personal disclosure avoidance, the aggregation measures also ensure that particularly sensitive areas such as schools, hospitals, and religious buildings cannot be singled out and re-identified.

Second, there are limitations in the data given the ongoing need to validate various dimensions of the data: Researchers have been aware of the population and temporal biases in the data. First, ownership is not equally distributed across all demographic groups, which we discussed earlier. Moreover, because we do not have information on individual users, only their home block groups, the representation of the sample within each block group may be unevenly distributed. In other words, we have corrected for geographic imbalances in population while assuming that, on average, the smart phone data is an unbiased sample within each block group. For instance, in addition to age, particular race, ethnicity, and class groups might be over- or under- represented in each block group. Future research will aim to better discover and adjust for these demographic variations with more advanced post-stratification techniques. Lastly, the temporal biases of smart phone data have been tested at locations such as polling stations^59^, sports games^60^, and airports^61^. However, given the lack of available ground-truth data covering longer periods and variation in activities, it remains difficult to comprehensively validate these data for temporal variation in their biases.

Third, our NERD measures observe potential for diverse race and ethnic interaction for observed stays within a *neighborhood*. That is, it measures all 24-hour activities in a neighborhood and encompasses the characteristics of the neighborhood that might draw people to the neighborhood. These could be the mixture of residents within the neighborhood, but it could be associated with schools, job centers, transportation infrastructure, public space, and other amenities in the neighborhood. In this sense, it is a neighborhood-centric - as opposed to an ego-centric - measure. Some users of this data product may also be interested in estimating the social exposure for residents of a particular tract, that is, the exposures across space of particular groups of people that share a common place of residence. Another possible future iteration of NERD could aggregate exposures across the daily trajectories of devices and associate these with the home tract.

Lastly, these measures have not accounted for the larger margins of error in the ACS 5-Year surveys compared to the decennial Census, which has been shown to overestimate aggregate segregation measures such as the racial dissimilarity index^62^, the information theory index, and the variance ratio index for income^63^. Because the decennial Census samples around 1 in 6 households in the United States, or about a 17% sample rate, and the ACS 5-Year’s sampling rate has ranged between 8% to 10%^63^, the ACS will generally underestimate the variation in the overall population compared to the decennial Census. While future iterations of NERD may incorporate margins of error in our estimates, a previous study using simulation techniques suggest that smaller population areas may over-estimate segregation^62^ and accounting for covariance between ACS observations leads to true margins that may vary with proportion of minority percentage^64^. Thus, we may expect these measures to under-estimate the actual experienced diversity to varying degrees, with smaller population and lower minority areas more likely to under-estimate diversity.

### Supplementary information

Supplementary Information

## Data Availability

This dataset was created using privately available human mobility data derived through cell phone GPS locations from MPA pings in Cuebiq’s Spectus Clean Room. While access to the original data is restricted, the data product and code underlying the methods is available on Open Science Framework^55^. Python 3.6 and SQL were used to generate the data outputs.
