# Supplementary material for: An experienced racial-ethnic diversity dataset in the United States using human mobility data
Source: Sci Data. 2024 Jun 17;11:638. doi: 10.1038/s41597-024-03490-y (PMC11183061; doi:10.1038/s41597-024-03490-y)
Supplement: Supplementary file 1 — Supplementary Information [file 41597_2024_3490_MOESM1_ESM.docx]

**Supplementary Information**


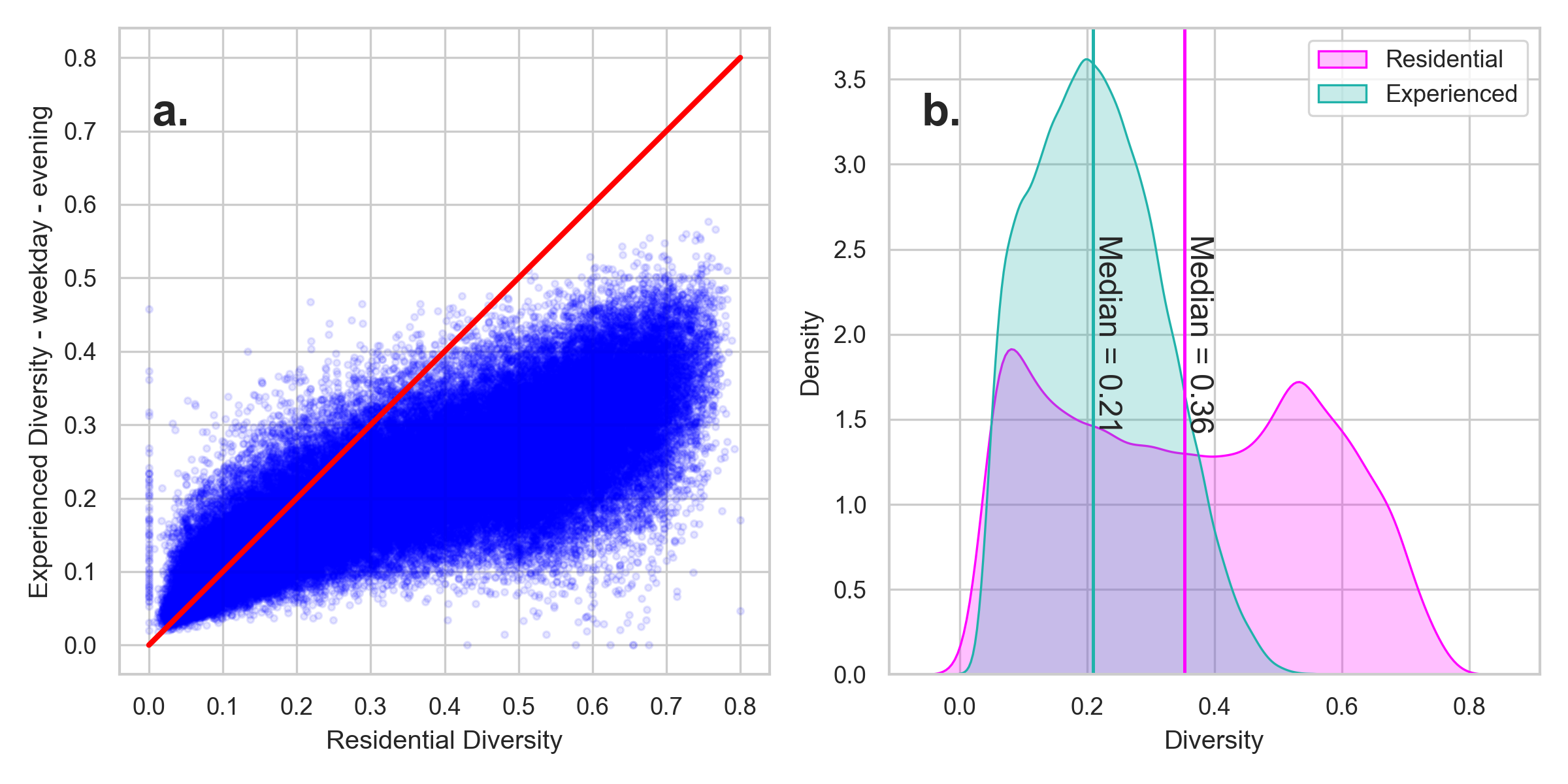


Figure S1: Residential and Experienced diversity scatterplot (a) and distribution (b) for weekday evenings


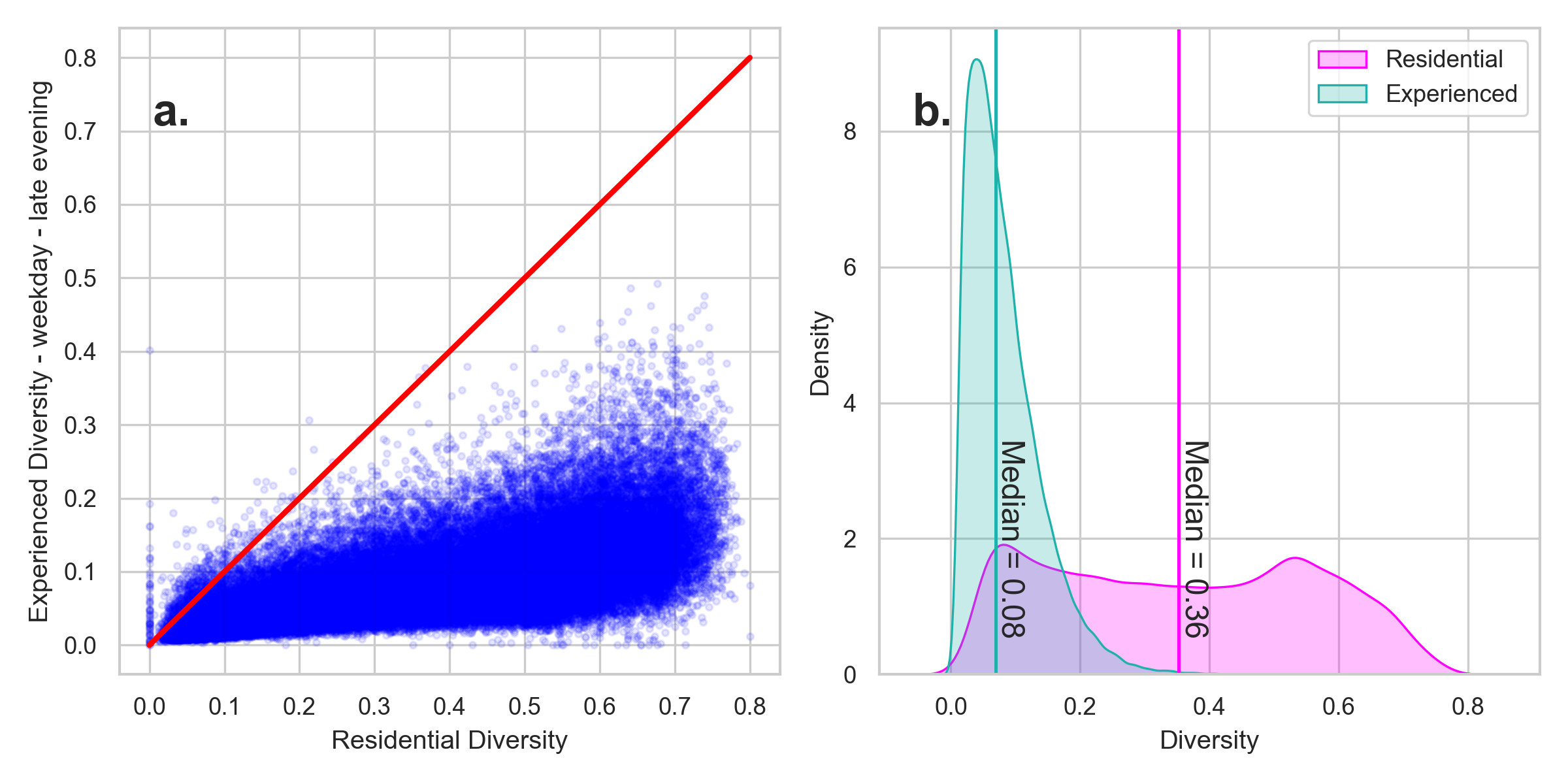


Figure S2: Residential and Experienced diversity scatterplot (a) and distribution (b) for weekday late evenings


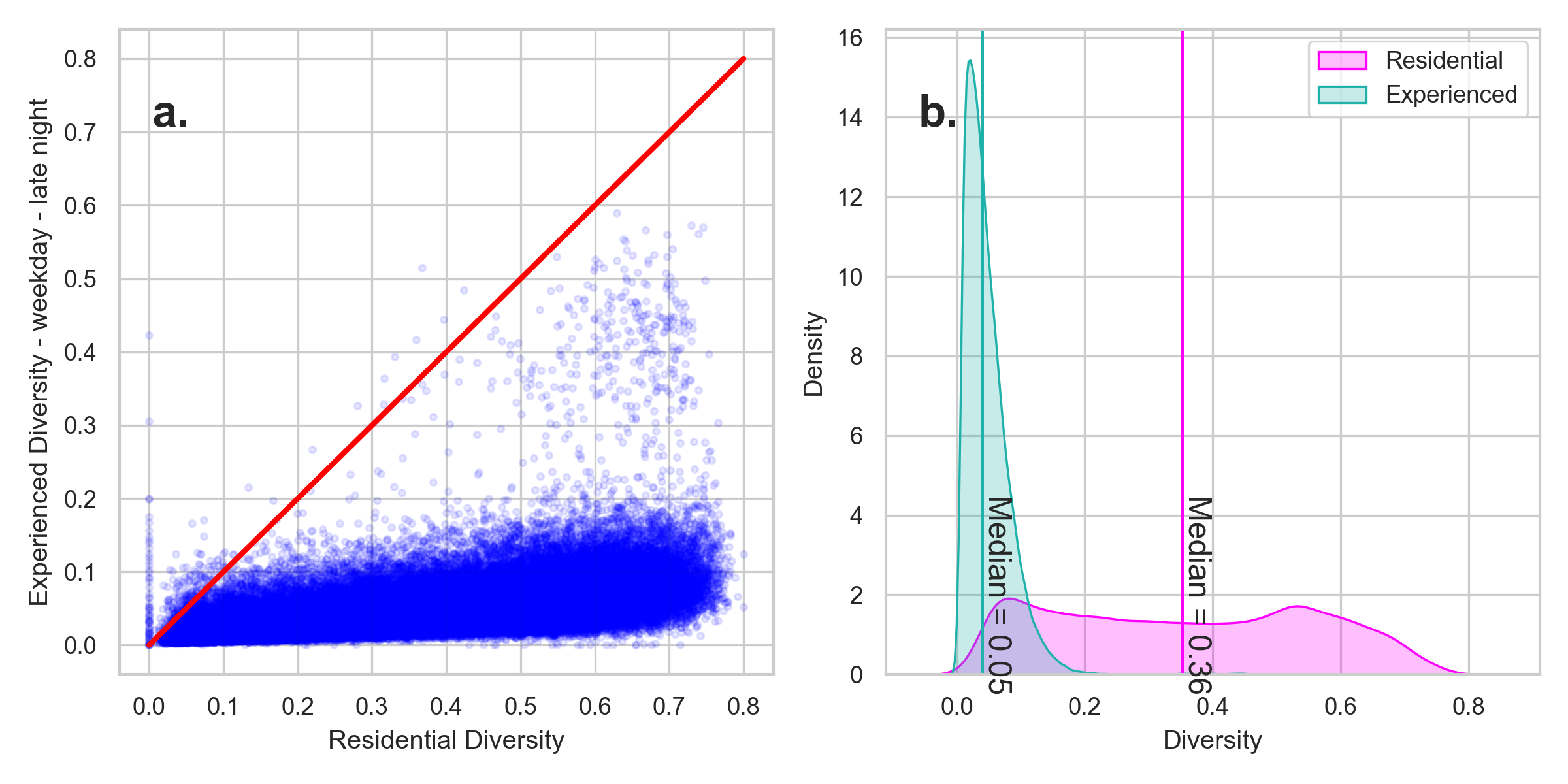


Figure S3: Residential and Experienced diversity scatterplot (a) and distribution (b) for weekday late nights


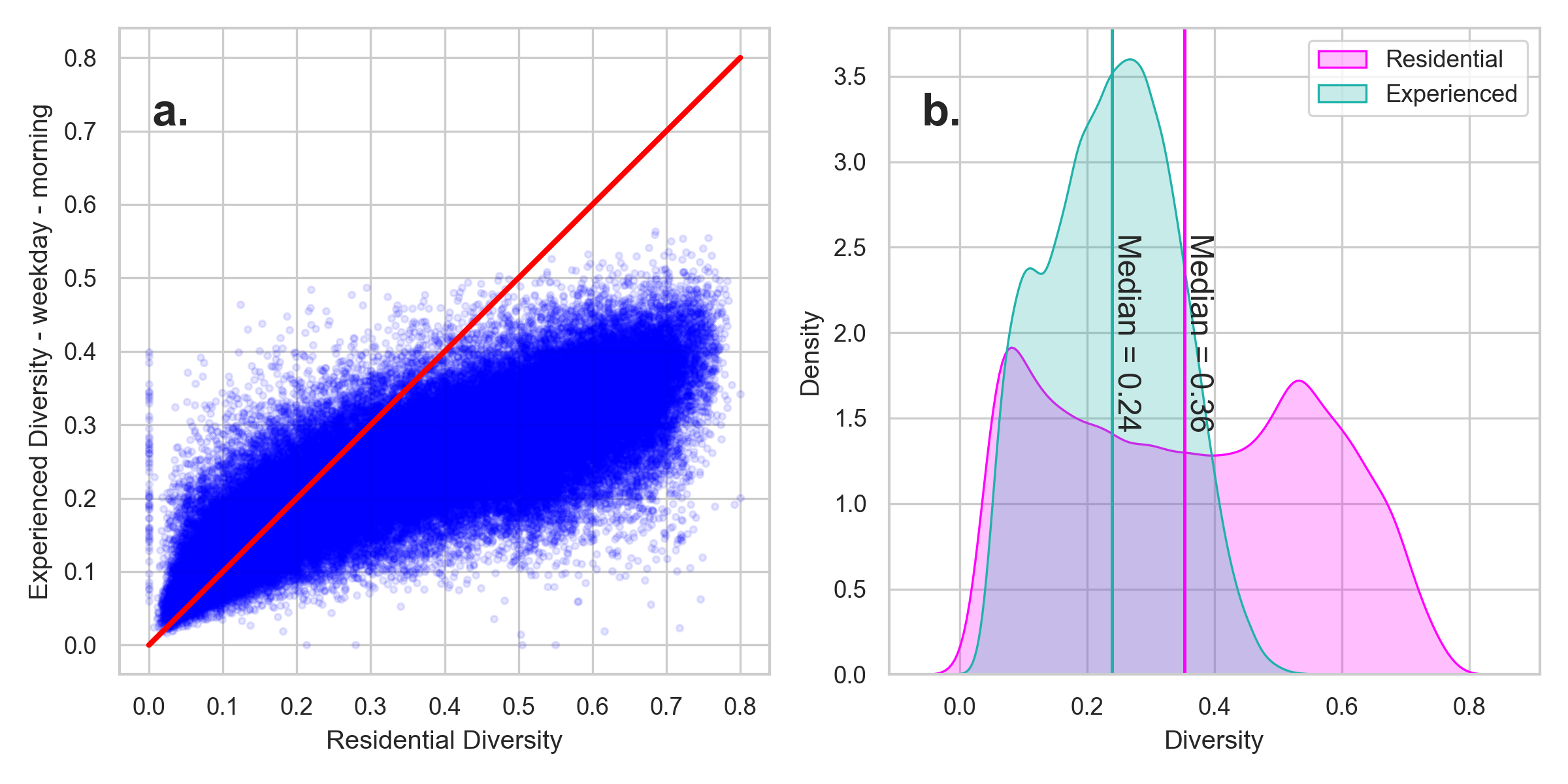


Figure S4: Residential and Experienced diversity scatterplot (a) and distribution (b) for weekday mornings


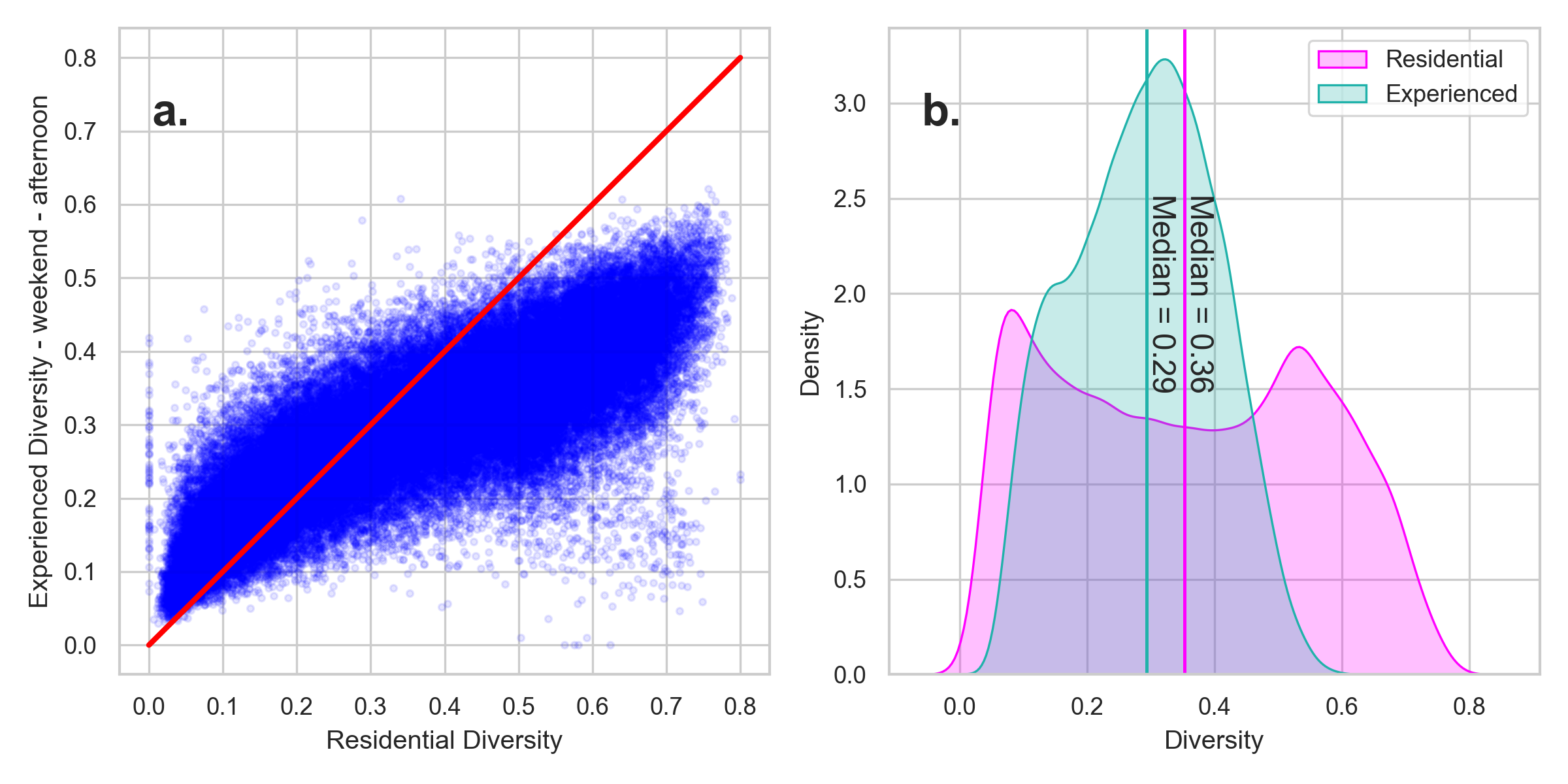


Figure S5: Residential and Experienced diversity scatterplot (a) and distribution (b) for weekend afternoons


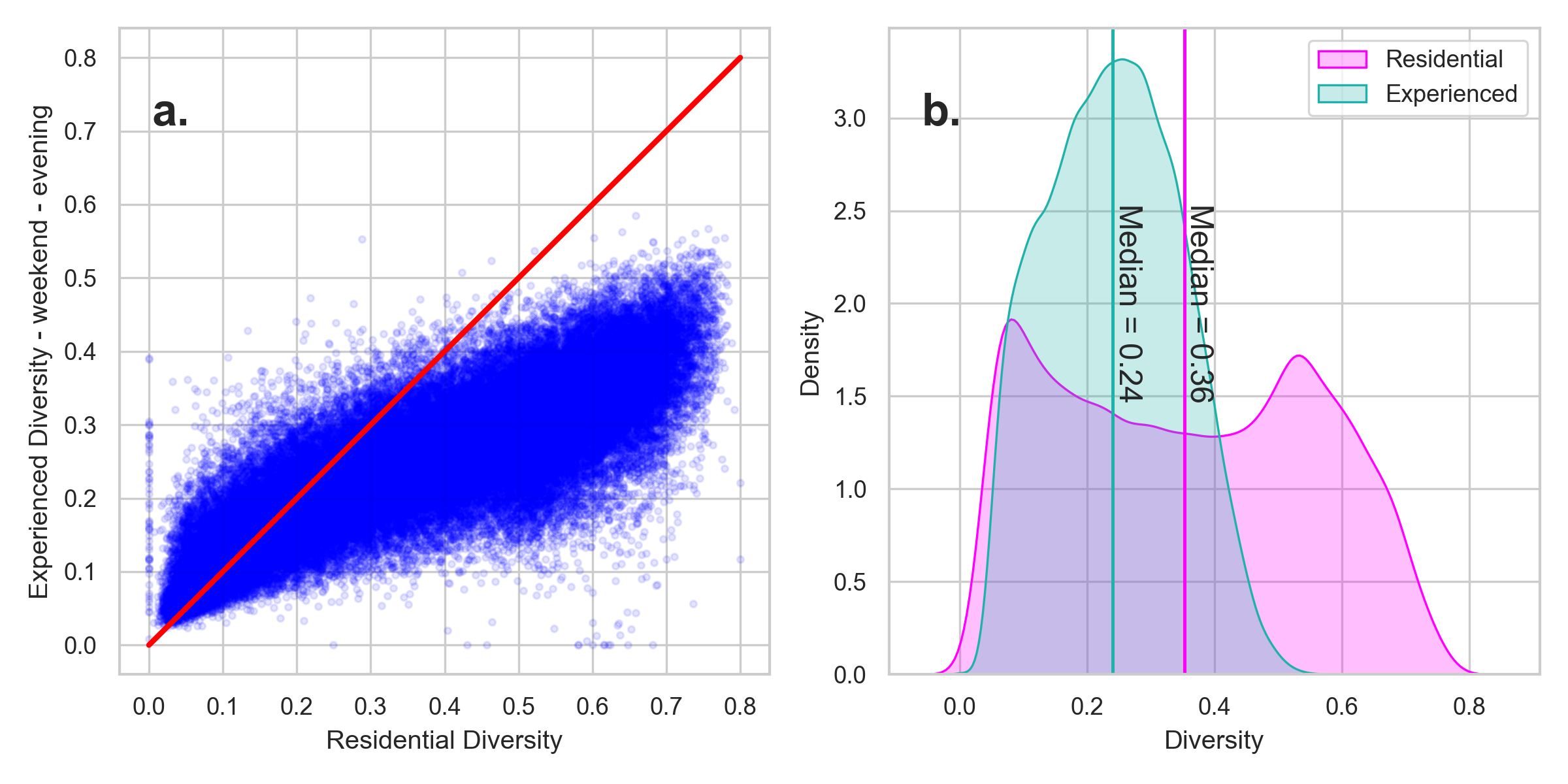


Figure S6: Residential and Experienced diversity scatterplot (a) and distribution (b) for weekend evenings


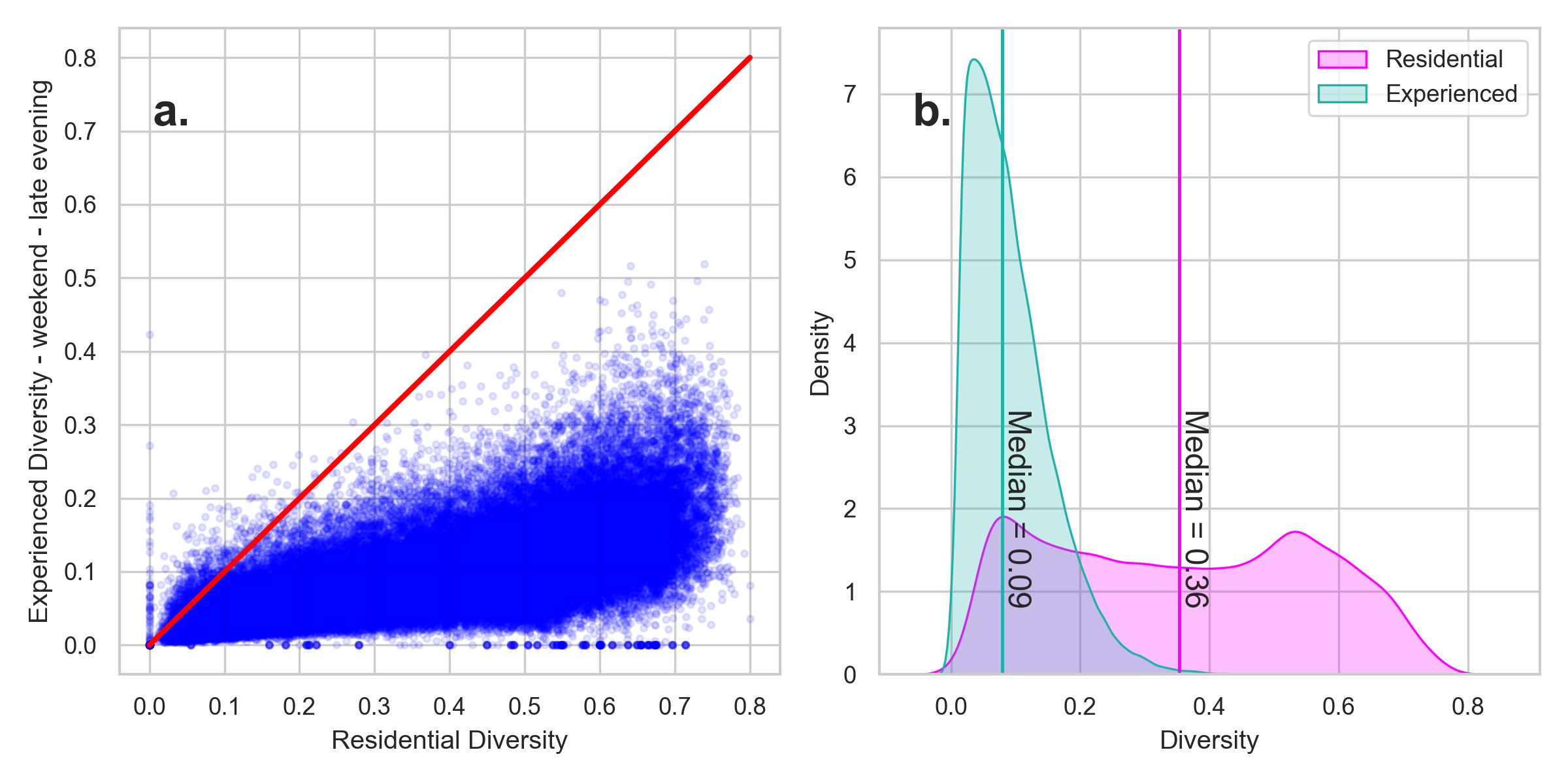


Figure S7: Residential and Experienced diversity scatterplot (a) and distribution (b) for weekend late evenings


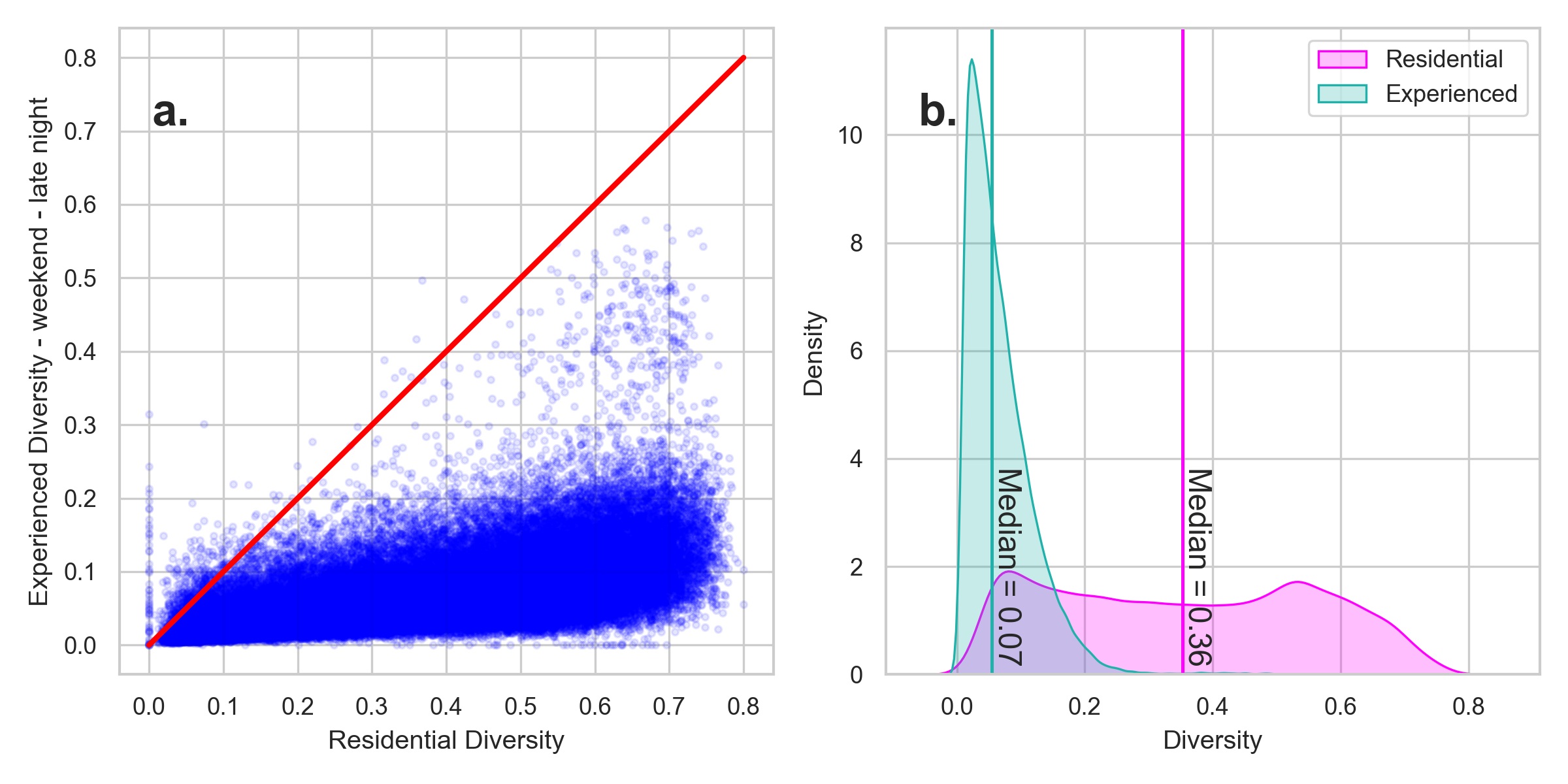


Figure S8: Residential and Experienced diversity scatterplot (a) and distribution (b) for weekend late nights


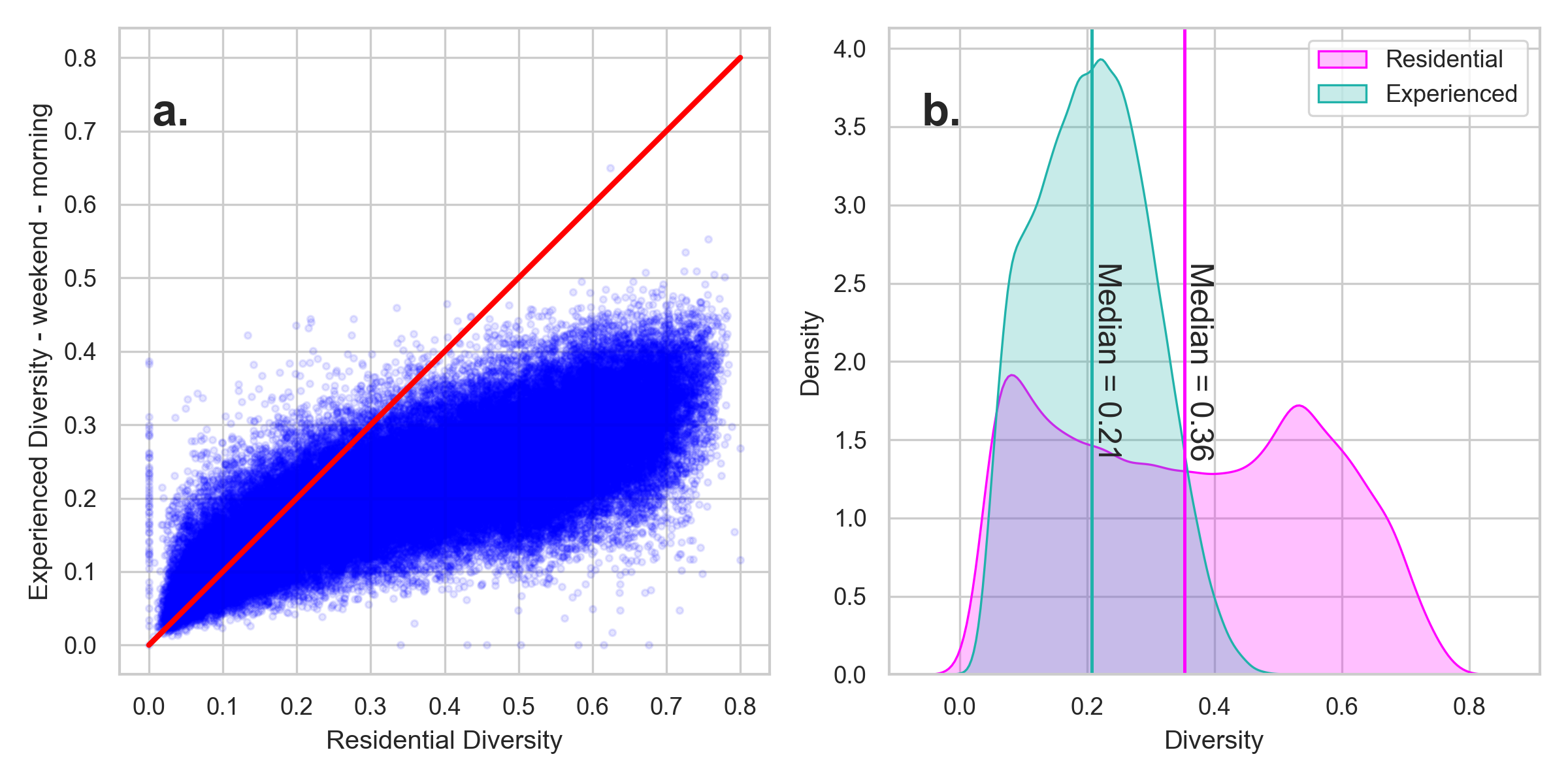


Figure S9: Residential and Experienced diversity scatterplot (a) and distribution (b) for weekend mornings
